# Diuretin and Diuresis—The Theoretical Side

**Published:** 1894-02-17

**Authors:** Benjamin Ward Richardson


### Diuretin and Diuresis.—The Theoretical Side.

By Sir Benjamin Ward Richardson, M.D., F.R.S.

Much attention has been paid of late to the action and therapeutical service of the substance called diuretin. Diuretin is the double salt of sodium salicylate and theobromine, containing fifty per cent. of the latter. The name of the drug is unfortunate for its scientific reputation. The name suggests that the substance, as a medicine, is in the strict sense nothing but a diuretic. It may have this property, but it certainly has no specific claim distinguishing it as universally of more worth than other bodies of the same class, and it has properties which the name does not altogether describe; to an extent, therefore, the term is deceptive and bears with it a sort of charlatanic quality, as if the drug did what no other drug would do, and as if it always succeeded though other diuretics might fail.

On myself, as on some other physicians, this impression led me at first to look upon the substance as a mere advertised product; but having learned from a brother physician, whom I knew to be a good observer, that he had found it to be of service in a case of suppression of urine with mitral obstruction, I determined to cast all prejudice aside and to investigate it carefully. I shall show in the sequel what conclusions I have been led to form on the matter, but before doing so I must devote a few moments to the study of the action of diuretin and diuretics from a physiological point of view.

It is first of moment to understand what a diuretic is and how it works. We say, in common phraseology, that a diuretic is a medicine that increases the flow of urine. In this sense water may be a diuretic, dieuresis being, after all, a question of degree. In a warm day abstinence from fluids, enforced or otherwise, causes a diminished flow of urine in healthy persons, accompanied with thirst; a free supply of water taken by the stomach alters the condition, and the flow increases; the water acts as a diuretic. In the healthy state of the kidney, and with a standard discharge, say of sixty fluid ounces a day, a varying quantity of watery fluid taken by the mouth modifies the standard discharge, and, absorbed in sufficient quantity can, unless there be some unusual elimination by the skin or by some other emunctuary, always be made to cause increase from the standard, and so far a diuresis. Certain substances added to water also increase the flow without reference to amount of fluid imbibed, and these substances, always limited as to the extent of their action, and always seemingly capricious as to their effect, are strictly diuretics.

Secretion of urine is a process of dialysis, and under purely natural conditions the prime factor is the salt urea. A soluble salt like urea acting on a colloidal fluid like the blood, or its albuminous serum circulating in the kidney, is a perfect factor for fixing the water of the colloid, and bearing it off by an excretory channel in the form of aqueous saline. Urea is the precise saline or crystalloidal substance wanted for the function. Hence urea plays a two-fold part; it not only conveys away the nitrogen of the tissues, but it acts as the crystalline factor in the vital dialysis of the kidney. In the absence of urea, or some precisely corresponding salt, there could be no excretion of urine; while in the presence of an excess of urea, or of a corresponding salt, there will, to a limited degree, be an excess of urine as of a diuretic acting by an increased dialysis.

If the kidney during its action were under no other influence than that of dialysis all would be simple and straight sailing; but this would not suit the varying moods and tenses of a living organism. Three other influences have therefore to be considered, namely: (1) The pressure of the blood by which the kidney is fed; (2) the nervous function over the minute blood-vessels; (3) the controlling power of the investing capsule. In thinking of diuretics, we must take all these points into consideration.

Some very soluble salines, like urea, potassium nitrate, ammonium nitrite, and sodium bicarbonate, are saline diuretics that sustain, or it may be intensify, the dialysing process; some increase the blood pressure, and, as it were, force the minute circulation of the kidney, digitalis seeming to be as good a specimen as can be desired of this order; some by reducing the nervous tension produce increase of blood in the kidney, and, if the dialysis be good, an increased flow, nitrite of ethyl, alcohol much diluted, and perhaps juniper being diuretics of this order. Lastly, some appear to work by a special action on the renal epithelium, producing what might be an increased discharge like the running of tears caused by a foreign substance impinging upon the conjunctiva.

By selecting our diuretics so as to meet these varying conditions, according to the character of the disease that leads to suppression of, or limitation of flow, we can arrive at something like precision in our prescription for a diuretic. Or, we can combine two or more of the series, so as to get a more certain and perfect action. In many cases it is most advantageous to combine any one of the three substances last-named with a saline, so that dialysis may go on unimpeded under an increased distribution of the blood.

But whatever we do in our attempts to increase the urinary flow, either by blood pressure, by nervous innervation, by irritation of the lining membrane, by enforced dialysis, or by two or more of these agencies in combination, we must never forget the controlling influence of the capsule. The capsule is not placed round the kidney simply to hold the organ in its place; it is there to limit the secreting boundaries of the organ. However much we may try to force the function, we cannot do more than the capsule will permit without injury to the texture of the cortical or medullary parts it encloses, and nothing can explain more forcibly a phenomenon we often witness, when, under what we consider the full swing of diuretic action, we suddenly are surprised by the occurrence of a suppression that obliges us to suspend our treatment.

Returning for one instant to diuretin, that compound ranks amongst those in which a feeble dialysing saline is combined with an alkaloidal body that has the power of acting on the renal epithelium, and as this leads to the clinical observations I have been able to make in regard to the practical value of the drug, they will form a text for my next communication.

